# esFont: A guided diffusion and multimodal distillation to enhance the efficiency and stability in font design

**DOI:** 10.1371/journal.pone.0333496

**Published:** 2025-10-09

**Authors:** Weijia Zhu, Xinjin Li, Jing Pu, Jin He, Jing Tan

**Affiliations:** 1 Department of Design, Zhijiang College of Zhejiang University of Technology, Shaoxing, Zhejiang, China; 2 Department of Computer Science, Columbia University, New York, New York, United States of America; 3 Department of Arts and Media, Sichuan Agricultural University, Yaan, Sichuan, China; 4 Department of Art Design, Zhejiang Gongshang University Hangzhou College of Commerce, Hangzhou, Zhejiang, China; 5 Department of Arts and Design, Mianyang Teachers College, Mianyang, Sichuan, China; Shanghai Maritime University, CHINA

## Abstract

Font design is an area that presents a unique opportunity to blend artistic creativity and artificial intelligence. However, traditional methods are time-consuming, especially for complex fonts or large character sets. Font transfer streamlines this process by learning font transitions to generate multiple styles from a target font. Yet, existing Generative Adversarial Network (GAN) based approaches often suffer from instability. Current diffusion-based font generation methods typically depend on single-modal inputs, either visual or textual, limiting their capacity to capture detailed structural and semantic font features. Additionally, current diffusion models suffer from high computational complexity due to their deep and redundant architectures. To address these challenges, we propose esFont, a novel guided Diffusion framework. It incorporates a Contrastive Language–Image Pre-training (CLIP) based text encoder, and a Vision Transformer (ViT) based image encoder, enriching the font transfer process through multimodal guidance from text and images. Our model further integrates deep clipping and timestep optimization techniques, significantly reducing parameter complexity while maintaining superior performance. Experimental results demonstrate that esFont improves both efficiency and quality. Our model shows clear enhancements in structural accuracy (SSIM improved to 0.91), pixel-level fidelity (RMSE reduced to 2.68), perceptual quality aligned with human vision (LPIPS reduced to 0.07), and stylistic realism (FID decreased to 13.87). It reduces the model size to 100M parameters, cuts training time to just 1.3 hours, and lowers inference time to only 21 minutes. In summary, esFont achieves significant advancements in both scientific and engineering domains by the innovative combination of multimodal encoding, structural depth pruning, and timestep optimization.

## 1. Introduction

As a core carrier of human civilization and a means of information transmission, text has long been associated with profound cultural and ideological connotations [1]. Fonts, as an important form of expression, not only affect the visual effect during communication, but also give texts emotional and artistic expression. However, font design is often considered a complex, time-consuming and labor-intensive task, as it requires designers to strike a balance between style consistency and aesthetics [2]. At the same time, existing methods generally have problems such as single model input, inability to fully extract font features that need to be transferred, large model calculation requirements, and complex models. In the early stage of the development of font transfer methods, like other image generation tasks, the main backbone network was based on generative adversarial networks. Reviewing the font transfer network based on Generative Adversarial Network (GAN) [3–5], often suffer from training instability such as mode collapse and non-convergence [6,7] due to their adversarial optimization process. A key issue is bias in the discriminator, where imbalanced decision boundaries cause the model to favor specific style features, limiting generalization to diverse font forms [8]. This can further lead to mode collapse, where the generator outputs only a narrow set of font variations, failing to reflect the full distribution of the target font style [9,10]. This can lead to inconsistent or poor quality of generated font styles. At the same time, if the design of the discriminator is biased, it will lead to mode collapse and fail to truly generate the target font image. Recently, the emerging large models have been used by more and more researchers for text, image and other processing tasks due to their large number of parameters [11].

As a prominent model in image processing, especially image generation, the diffusion model has attracted widespread attention since it was proposed, and has also been used in font transfer tasks. Kondo et al. [12] introduced font style interpolation using diffusion models, effectively modelling smooth transitions between styles but lacking semantic guidance due to reliance solely on visual inputs. He et al. [13] proposed Diff-Font for robust one-shot font generation based on diffusion models. While effective at single-style font synthesis, its single-modal, image-only input approach limits semantic detail representation, impacting the variety of generated styles. VecFusion [14] applies diffusion models to generate vectorized fonts, aiming for precise control over vector outputs. However, the method struggles to capture stylistic nuances, again due to its exclusive visual input. Chen et al. [15] presented CD-Font, using conditional diffusion models with disentangled guidance, enhancing style control but still constrained by single input modality, limiting overall expressive detail. DP-Font by Zhang et al. [16] incorporates physical information networks within diffusion models for Chinese calligraphy fonts, improving structure realism but at the expense of significant computational complexity. These prior approaches share two critical limitations: the network that relies on a single input cannot capture the diversity and details of the input font, resulting in a single-generated font style. At the same time, the generation process of the diffusion model is very complicated, which will lead to low efficiency and low portability in the generation process. Therefore, this paper aims to build an efficient, stable and controllable font style transfer model.

Through the understanding and analysis of existing methods, it is obvious that image and text inputs have their own advantages. Image input can directly provide contour and structural information, while text input directly provides semantic and detailed supplements. The semantic details are not used to describe the meaning of Chinese characters, but the structural visual attributes of Chinese characters themselves, including global style, stroke characteristics and typesetting layout. Compared with traditional alphabetic characters, Chinese fonts have more strokes and more complex structures. Therefore, adding semantic details to the structure can provide additional supplements in the generation, thereby improving the consistency of font structure and style. Currently, these methods only use one of the inputs and ignore the supplement of the other input. If only visual features are used, the model can only imitate the outline of the reference font and it is difficult to adjust the stroke details; if only semantic features are used, the model can only imitate the description of the reference font but cannot provide geometric details such as stroke layout. Fusion of the two into a latent vector Z allows the diffusion process to be guided by both stroke details and font style, thus achieving a balance between structural fidelity and semantic consistency. Therefore, we propose a diffusion model that combines image and text inputs. Image input provides structural and contour information for fonts, and text input provides semantic details for fonts. The combination of the two enhances the stability and quality of the diffusion model. At the same time, depth pruning and time step optimization methods are introduced in the diffusion model to reduce the model size and training and inference time.

Specifically, we use Vision Transformer (ViT) [17] to encode the reference font image and extract the contour and structural information of the font. The global self-attention mechanism in ViT captures long-range dependencies across strokes, allowing it to learn more comprehensive geometric structures than a conventional CNN. At the same time, we use Contrastive Language–Image Pre-training (CLIP) [18] text encoder to encode the text information and analyze the semantic information and detailed supplement of the text input. Having been jointly pre-trained on a large-scale image–text corpus, the CLIP text encoder produces semantic vectors that are intrinsically aligned with visual features and supply finer stylistic cues. Subsequently, the encoded image features and text features are fused into a latent vector Z through MLP. This latent vector Z is added in the diffusion process to play a guiding role in each stage, thereby effectively improving the generation quality and stability. After fusing the latent vector Z, the original deep computational structure of the diffusion model will lead to redundant information transmission, thus reducing computational efficiency. At the same time, we observed that the shallow depth provides the overall framework for font transfer, and the deep depth provides details for the font, and there is less need for detail optimization. In order to reduce information redundancy in the diffusion process and improve information utilization, we choose to introduce depth pruning, discard the redundant layers, and reduce the computational cost. However, the information loss caused by depth pruning will be further amplified as the original time step is too long, which will affect the performance of the model. At the same time, we also observed that the contributions of early and late time steps to font generation are not equal. Therefore, in order to reduce the impact of information loss and skip time steps with low contribution, we introduce a time step optimization method. Time step optimization can adjust the distribution of time steps in the diffusion process, concentrate the number of computational steps on the steps that have a greater impact on the generation quality, and minimize the impact of information loss caused by deep jump shearing.

In summary, our method combines the advantages of image input and text input, uses multimodal input to accurately control the generation process of the diffusion model, and greatly improves the computational efficiency of the model through pruning and time step optimization. The Structural Similarity Index (SSIM) [19] is 0.91, the Root Mean Square Error (RMSE) is 2.68, the Perceptual Image Patch Similarity (LPIPS) [20] is 0.07, and the Fréchet Inception Distance (FID) [21] is 13.87, which are improved compared with existing methods. At the same time, the number of parameters is reduced to 100M, the training time is shortened to 2 hours, and the inference time is only 10 minutes, which provides a new idea for stable and efficient font style migration.

The primary contributions of this paper can be summarized as follows:

1. We proposed a multimodal input Diffusion model for font transfer tasks. We introduced a multimodal input strategy of text and image fusion in the Diffusion model, embedded the CLIP text encoder and ViT image encoder into the diffusion process, and provided rich feature representations for font style migration through the guidance of text and pictures, thereby improving the stability and diversity of generated fonts.
2. We proposed deep clipping and time step optimization techniques in the Diffusion model to reduce the diffusion depth and diffusion steps in the model, thereby greatly reducing model parameters and computing resource requirements. In addition, it reduces computational costs and enhances the potential for model transferability.
3. According to the experimental results, our proposed method outperforms other GAN-based and diffusion model-based approaches in terms of character details generation, similarity with the target font, and generation stability. It achieves the best performance in SSIM, RMSE, LPIPS, and FID, while the number of parameters, training time, and inference time are minimized.

The following article is arranged as follows: Section 2 introduces the related work of this article, including the introduction of the diffusion model and the compression of the diffusion model. Section 3 introduces our proposed method, describes in detail the diffusion process of introducing image input and text input, as well as the pruning and optimization of the diffusion process. Section 4 conducts qualitative and quantitative analysis on the dataset and shows the improvement results of the model. Chapter 5 summarizes this article.

## 2. Components

### 2.1 Diffusion model

The denoising diffusion probability model was improved by Nichol A Q [22]. It is a deep learning model built upon probabilistic generation principles. In recent years, due to its powerful generation ability and stability, the diffusion model has received increasing attention in most image generation tasks, especially font transfer tasks. The diffusion model involves a process where noise is gradually added to the input image, transforming it into a Gaussian distribution, followed by a step-by-step denoising process via learning. Through this process, the input image is converted into a high-quality target image, thereby achieving data regeneration. Its working principle can be understood as two stages: the noising process and the denoising stage. The first is the forward denoising process. For a given input data $x_{\text{0}}$ , a complete Markov chain is used to gradually add noise to the input to obtain the noise data $x_{t}$ at each time step. When t increases to the terminal step T, $x_{T}$is close to pure Gaussian noise; then the model will gradually denoise in the opposite direction and restore $x_{T}$ to a clean target image. Its forward transfer probability is defined as:


$$q(x_{t}|x_{t-1})=N(x_{t};\sqrt{1-\beta_{t}}x_{t-1},\beta_{t}I)$$ (1)

Where $\beta_{t}$ is the noise coefficient that changes with time step t. After obtaining the complete noise data $x_{t}$, a denoising process is required. The denoising process is to denoise the noise data in the noise process by learning a neural network with parameters $p_{\theta }\left(x_{t-1}|x_{t}\right)$, and finally gradually restore the target data $x_{\text{0}}$ from pure noise $x_{T}$:


$$p_{\theta }(x_{t-1}|x_{t})=N(x_{t-1};\mu_{\theta }(x_{t},t),\Sigma_{\theta }(x_{t},t))$$ (2)

Among them, $\mu_{\theta }$ and $\sum_{\theta }()$ are the mean and variance continuously calculated by the model during the learning process.

Although multimodal text and image inputs have improved the generation quality and stability of the diffusion model, they have also brought problems with computational efficiency. The deep structure in the diffusion model causes information redundancy in the newly added input. After the redundant information is transmitted at multiple levels, it will inevitably lead to performance degradation, and it is difficult to maximize the information utilization. Therefore, the model still needs depth pruning to optimize the structure of the diffusion model and improve information utilization.

### 2.2 Font style transfer

In recent years, font style transfer has been extensively studied using GAN and Diffusion models. Xie Y. et al. [23] proposed a new deformable generative network based on generative adversarial network. It introduced a feature deformation skip connection into the generator, which is used to predict the displacement map of the input and pass this map into the deformable convolution for low-level feature map. Although this design enhances the feature extraction of the input image font, the generation and adversarial training methods are susceptible to instability issues during the generation process. As a result, the quality of the generated font images is often inconsistent, especially when dealing with complex font styles. At the same time, the generative adversarial network is prone to mode collapse, that is, the generation quality is very poor, but the generator cannot update parameters due to the weak performance of the discriminator. Kondo T. et al. [12] proposed an improved diffusion process. It changes the original diffusion process of denoising from pure noise, and instead uses the input reference font and the target character to be superimposed and added to the denoising process, that is, gradually denoising from noise to artificial images. This process allows the diffusion model to fully learn the outline and structure of the font to a large extent. However, the lack of text description will lose a lot of details about the font, such as the semantic accuracy of the target character, which refers to the preservation of the character’s intended identity and structural integrity during generation. Rombach, R. [24] proposed a stable diffusion model from text to image. It accepts descriptions of common fonts and descriptions of new fonts to complete the style transfer task from common fonts to newly designed fonts. The text description of the font is important, but when there is a lack of image input, the lack of font structure and outline information has a great impact on the quality. Cheng S. I. et al. [25] proposed a diffusion model that combines stroke images and sketch images as conditions to generate font images from hand-drawn images. Zhang L. et al. [26] proposed a diffusion model that introduces an additional conditional control module so that it only fine-tunes the control model to perform specific font transfer tasks. Zhu Y. et al. [27] proposed a diffusion model that takes three inputs, image, text and style, as conditions to generate specific text images. He H.et al. [13] proposed a diffusion model that takes character content and style as conditions, and uses these conditions to generate corresponding character images. Through the analysis and organization of the above research, we have been inspired. Images usually include the necessary outline and structure information of fonts, while text information can provide some additional details for the generated fonts. By combining the two, they can serve as conditional inputs to steer the generation process of the diffusion model, thereby enhancing the quality of the generated output. Hence, this paper introduces an improved diffusion model that integrates both image and text guidance to direct the model in generating high-quality migration fonts.

### 2.3 Model compression in diffusion models

In recent studies, many researchers have focused on model compression in diffusion models. Lu C. et al. [28] proposed a method to treat the diffusion probability model as a diffusion ordinary differential equation (ODE). By applying variable transformation, it can simplify the solution of the equation in the diffusion process to an exponentially weighted integral in the network, thereby achieving an acceleration process in the diffusion process. Jolicoeur-Martineau A. et al. [29] proposed a carefully designed Stochastic Differential Equation (SDE) solver. It can customize the process for the score-based diffusion model one by one with adaptive step size, and can generate high-quality samples with only two score function evaluations. The addition of ODE and SDE solvers has accelerated the diffusion process, but in the font transfer task, it affects the diversity of font generation. At the same time, the addition of additional modules requires more training of the model, which also increases the computational overhead of the model. Li Y. et al. [30,31] proposed a method to strengthen the distribution distillation process by introducing regularization to the classifier-free model. In the distillation process, it adds additional regularization terms to the learning objective to guide the training direction. The teacher-student distillation model can effectively reduce the model size, but the control of model quality becomes complicated. The above exploration of model compression is exciting, but there are still problems such as the need for additional training and guided training in the font transfer task. The deep clipping and time step optimization introduced in this article can be implemented directly in the diffusion model without adding additional modules. At the same time, the original model continues to be used without additional guided training. These two improvements can simplify the diffusion process and obtain the same generation quality.

## 3. Methods

### 3.1 Conditional diffusion model

**Fig 1:**
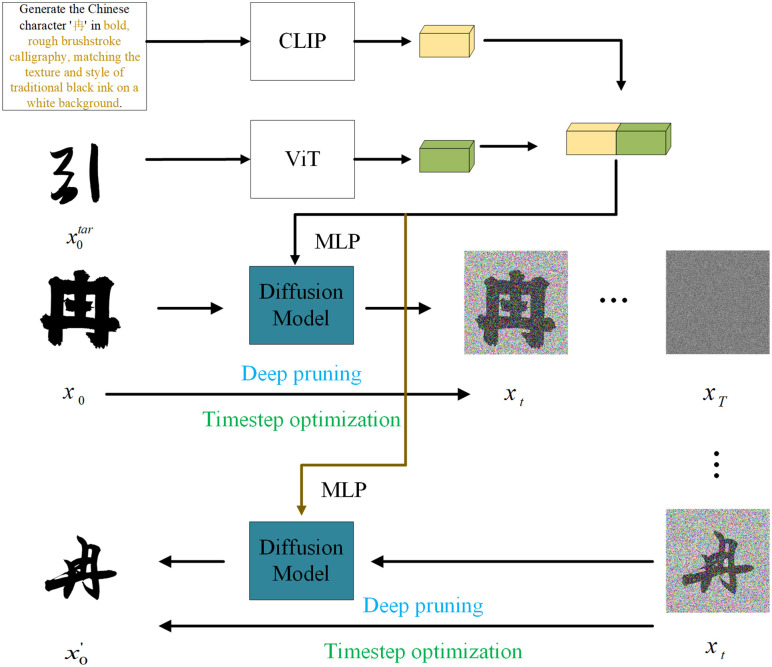
The flowchart of the large model for font style transfer. The model input includes image input and text input, which are fused into a latent vector to guide font generation. At the same time, deep clipping and time step optimization are introduced to speed up model generation.


In this chapter, we will introduce the details of the proposed framework. The main process of this paper is shown in Fig 1. As shown in the figure, the target font image is directly input into the diffusion process. At the same time, it is encoded and linked with the text input separately, and then encoded into a latent vector z through MLP. Among them, $x_{\text{0}}^{tar}$ is a random glyph in the target font. Since only the font style of the reference font is provided, usually only one is selected to prove that the model can learn the font style from very few reference fonts. $x_{\text{0}}$ is the source font, which provides a complete skeleton of the font style and constrains the generated result to maintain the character structure. Since it only provides the skeleton structure of the font, its font style is usually ordinary, which makes it easier for the model to learn the basic structural information of the characters. $x_{\text{0}}^{\prime }$ is the predicted glyph after reverse denoising of the diffusion model, which contains the font style of the reference font and the glyph of the source font. During training, the loss is calculated for the glyph corresponding to the target font $x_{j}^{tar}$. Specifically, the input font image $x_{\text{0}}$ is extracted through ViT to extract high-dimensional features, capturing the structure and contour information of the font, including the font stroke curve, stroke thickness and alignment. In the process of extracting visual features, ViT relies on its multi-layer self-attention mechanism to integrate the local information of the image with the global context information to ensure that the captured features can accurately reflect the overall style and local details of the font. ViT divides the input image into a series of fixed-size image blocks, linearly maps them to a high-dimensional space, and then generates a visual feature representation through a self-attention mechanism. The feature extraction process for $x_{\text{0}}$ is described as:

$$v=ViT(x_{\text{0}})=f_{transformer}(f_{patch}(x_{\text{0}}))$$ (3)

Among them, $f_{patch}$ represents the segmentation and linear projection operation of the input, $f_{transformer}$ represents the multi-layer self-attention mechanism, and v is the visual feature vector extracted by ViT. At the same time, the target font style text description $x_{text}$ is feature encoded by the CLIP text encoder. CLIP first segments the input and maps these segments to the word vector space, and then uses the Transformer structure to capture the semantic relationship between sentences. After these processes, the text embedding vector is generated, which is described as:


$$t=\text{CLIP}(x_{text})=g_{transformer}(g_{embed}(x_{text}))$$ (4)

Among them, $g_{embed}$ is the word embedding operation, $g_{transformer}$ is the Transformer encoding module, and t is the generated text feature vector. Subsequently, the outputs v and t of ViT and CLIP are connected into a comprehensive feature representation. The feature connection is to ensure that the visual and semantic information can be effectively integrated, where the visual features contain the structural information of the font, and the semantic information contains the font style direction. Finally, the latent vector z is generated by the multi-layer perceptron:


$$\begin{array}{cc} & z=\text{MLP}([v;t])=h_{\text{mlp}}([v;t])\\ & h_{\text{mlp}}^{\left(i\right)}(x)=\sigma \left(W_{1}^{\left(i\right)}x+b_{l}^{\left(i\right)}\right) \end{array}$$ (5)

Among them, [v;t] represents the concatenated vector of the two, $h_{mlp}$ is a multi-layer perceptron module consisting of a fully connected layer and an activation function σ, where W and b are the weights and biases of the fully connected layer. In our task, the model sets the number of MLP layers to 2. The one-layer model is limited by the difficulty in effectively capturing the cross-modal interaction between vision and semantics, while the three-layer model is prone to overfitting and increases computational overhead. The two-layer structure achieves the best balance between the two. This two-layer MLP concatenates, fuses, and nonlinearly maps the style vector v extracted by ViT and the semantic vector t output by CLIP to a compact latent conditional vector z. Through feature integration and dimensionality reduction, it ensures that the downstream diffusion model can simultaneously utilize structural and semantic information, thereby generating a target font with high detail accuracy, consistent style, and stability.

In the standard diffusion model, the noising and denoising processes are expanded as (1) and (2), and are explicitly expressed by constructing ϵ~𝒩(0,1) as:


$$x_{t}=\sqrt{\beta_{t}}x_{\text{0}}+\sqrt{1-\beta_{t}}\epsilon ,$$ (6)

Equation (1) gives the single-step conditional distribution $q(x_{t}|x_{t-1})$ of the forward diffusion, which shows the result of scaling the previous state $x_{t-1}$ and superimposing Gaussian noise $\beta_{t}$ with variance at each time step. Recursively expanding this series of Markov chains and incorporating all noise terms yields the closed form of Equation (6). Therefore, Equation (6) is completely consistent with Equation (1) in the definition of noise schedule and random variables, except that the step-by-step injection is rewritten as a one-time sampling. This equivalence ensures that the statistical assumptions of the forward process are unchanged. After generating the latent vector z, it is added to the noising and denoising process. Therefore, the process is updated as:


$$x_{t-1}=\frac{\text{1}}{\sqrt{\beta_{t}}}(x_{t}-\frac{1-\beta_{t}}{\sqrt{1-{\overset{―}{\beta }}_{t}}}\epsilon_{\theta }(x_{t},t,z))+\sigma_{t}z,$$ (7)

Among them, $\epsilon_{\theta }\left(x_{t},t,z\right)$ is the noise component predicted by the model, ${\overset{―}{\beta }}_{t}$ is the accumulated scheduling parameter, and $\sigma_{t}z$ is the extended random noise term. Formula (7) has two key extensions based on the classic denoising formula (as shown in Formula (2) in Chapter 2): First, the prediction of noise in the original formula only depends on time step t and the image $x_{t}$ at time step t. However, in the improved model, the latent vector z obtained by MLP fusion is explicitly added to the input of the noise prediction network ϵθ, so that the network can use visual and semantic features for denoising and provide more guidance for font generation. Secondly, a random compensation $\sigma_{t}z$ is added outside the extension term, so that z can not only guide the prediction of noise, but also affect the generated distribution at each step, thereby the guidance of strengthening z on the generated distribution and improving sample diversity. In the extension term, when z=0 or $\sigma_{t}=0$, Formula (7) degenerates into the original reverse process, thereby ensuring compatibility with the standard DDPM framework. After introducing the latent vector z containing visual and semantic information, the loss function should also be improved to adapt to the new conditional generation process. The loss function is expressed as:


$$L=E_{x_{\text{0}},\epsilon ,t}\left[∥\epsilon -\epsilon_{\theta }\left(x_{t},t,z\right){∥}^{\text{2}}\right]$$ (8)

Among them, ϵ is the real noise. Such a loss function can ensure that the diffusion model not only focuses on noise removal, but also gradually guides the generation results to the target style font.

Although multimodal text and image inputs have improved the generation quality and stability of the diffusion model, they have also brought problems with computational efficiency. The deep structure in the diffusion model causes information redundancy in the newly added input. After the redundant information is transmitted at multiple levels, it will inevitably lead to performance degradation, and it is difficult to maximize the information utilization. Therefore, the model still needsdepth pruning to optimize the structure of the diffusion model and improve information utilization.

### 3.2 Depth pruning

**Fig 2:**
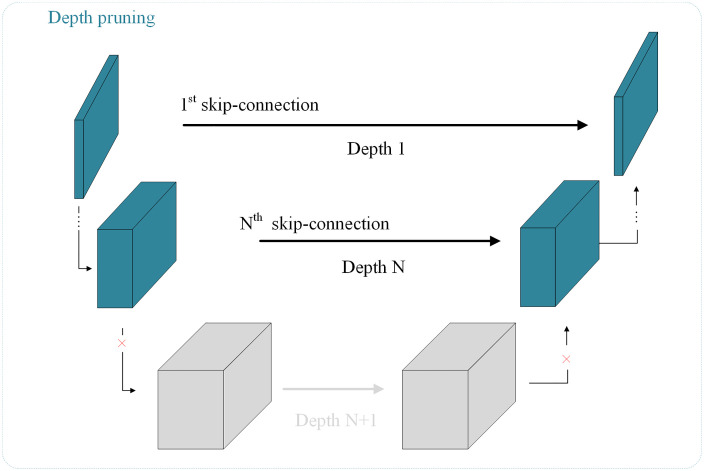
Depth pruning can effectively reduce the complexity of the model. The depths less than N are used, while the depths greater than N are dropped.

In the font transfer task, a diffusion model guided by text and images generates a font image with a given style. In the diffusion model of this paper, the backbone network uses U-Net, which is a U-shaped multi-layer convolutional network. It encodes and decodes the input during the noising and denoising process to achieve the noising and denoising operations. This is often complicated and lengthy due to the multi-layer design. In the U-Net architecture, in the shallower layers, the font layout is relatively clear, so these layers are mainly responsible for extracting and generating the overall structure of the font image, including shallow features such as contours and positions. In the deeper layers, the font layout is gradually noisy, and more detailed details are revealed, including the strokes and spacing information of the font, so these layers are mainly responsible for extracting and generating detailed features. Font images are different from conventional image generation. They do not contain a large number of generated details, contours and positions. A small part contains details of strokes and spacing, and most of them are blank. Therefore, cutting off the deep layers of U-Net will not have much impact on the output quality, because most of the structure and details of the font are in the shallower layers. As shown in Fig 2, we set a threshold in U-Net and directly discard the parts with depth higher than the threshold, while retaining the layers within the threshold. This not only improves the denoising speed of the model, but also maintains the generated quality.

Although depth pruning effectively reduces information redundancy and computational cost, the reduction in depth also leads to a reduction in effective information. In the diffusion time step, the distribution of the original time step will further amplify the information loss, resulting in performance degradation. Therefore, the model still needs time step optimization to optimize the model structure, reduce the impact of information loss, and improve time step efficiency.

### 3.3 Timestep optimization

In the font transfer task, the impact of time steps on model generation is not the same, that is, different time steps play different weighted roles in the font image generation process. The early time steps are mainly responsible for generating the overall framework and layout of the font, which mainly includes the outline and position of the font characters, and is a relatively important stage in the generation process. In the late time steps, it is mainly responsible for the fine adjustment of the style, which mainly includes the adjustment of stroke details, the adjustment of stroke spacing in characters, and the adjustment of texture details. Therefore, in the overall time step, the overall framework is the main feature in the early generation step, while the marginal benefit of the late time step is relatively low, and the late time step can be optimized. In order to generate efficiently, we use the γ curve formula to generate the time step sequence, and its main steps are:


$$F_{t}(\gamma ,n)=T\cdot t^{\gamma }$$ (9)

**Fig 3:**
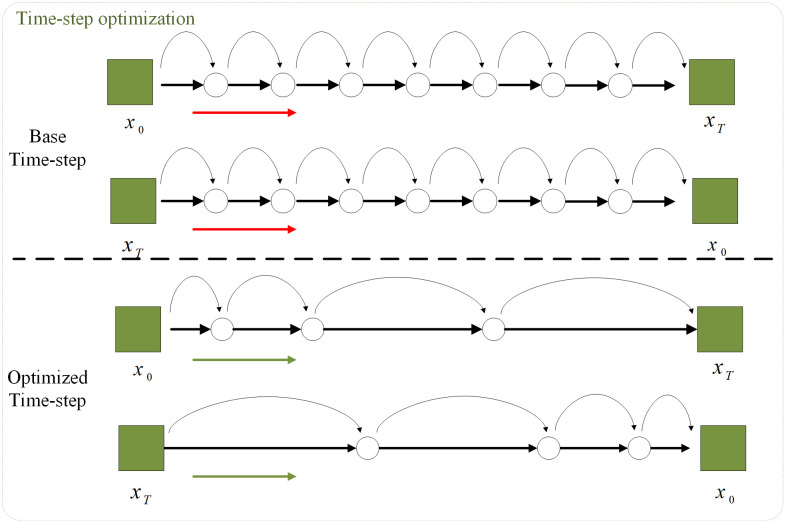
Time step optimization can greatly improve the efficiency of training and inference. In the process of adding noise and denoising, giving priority to the time steps that are more important for generation can improve the computational efficiency.

Among them, γ>0 is used to control the distribution of time steps. When γ>1, the time steps are mainly concentrated in early time steps. When 0<γ<1, the time steps are mainly concentrated in late time steps. In order to further simplify the generation process, γ=1.5 is directly fixed, making the overall production process more biased toward early time steps, as shown in Fig 3. Under the fixed time step optimization scheme, the generation process can quickly generate the outline of the font in the early time step, and add details in a certain later time step, significantly reducing redundant calculations, improving the generation efficiency and outputting at the same time High quality font images.

The introduction of timestep optimization is crucial to improving generation quality and reducing computational costs. It improves the performance of the model by changing the distribution of the original timesteps, reducing redundant calculations, and reducing the impact of information loss on the model. However, relying solely on timestep optimization cannot directly improve model performance, especially in the case of information redundancy and structural redundancy. Overall, the multimodal fusion input, depth pruning, and timestep optimization in our model are an inseparable whole. Among them, multi-module fusion input provides structural and semantic guidance, depth pruning optimizes the information transmission process, and timestep optimization balances the quality and efficiency of the generation process. We organically combine these three together to fundamentally improve the performance and efficiency of font transfer.

## 4. Results

### 4.1 Experimental design

**Fig 4:**
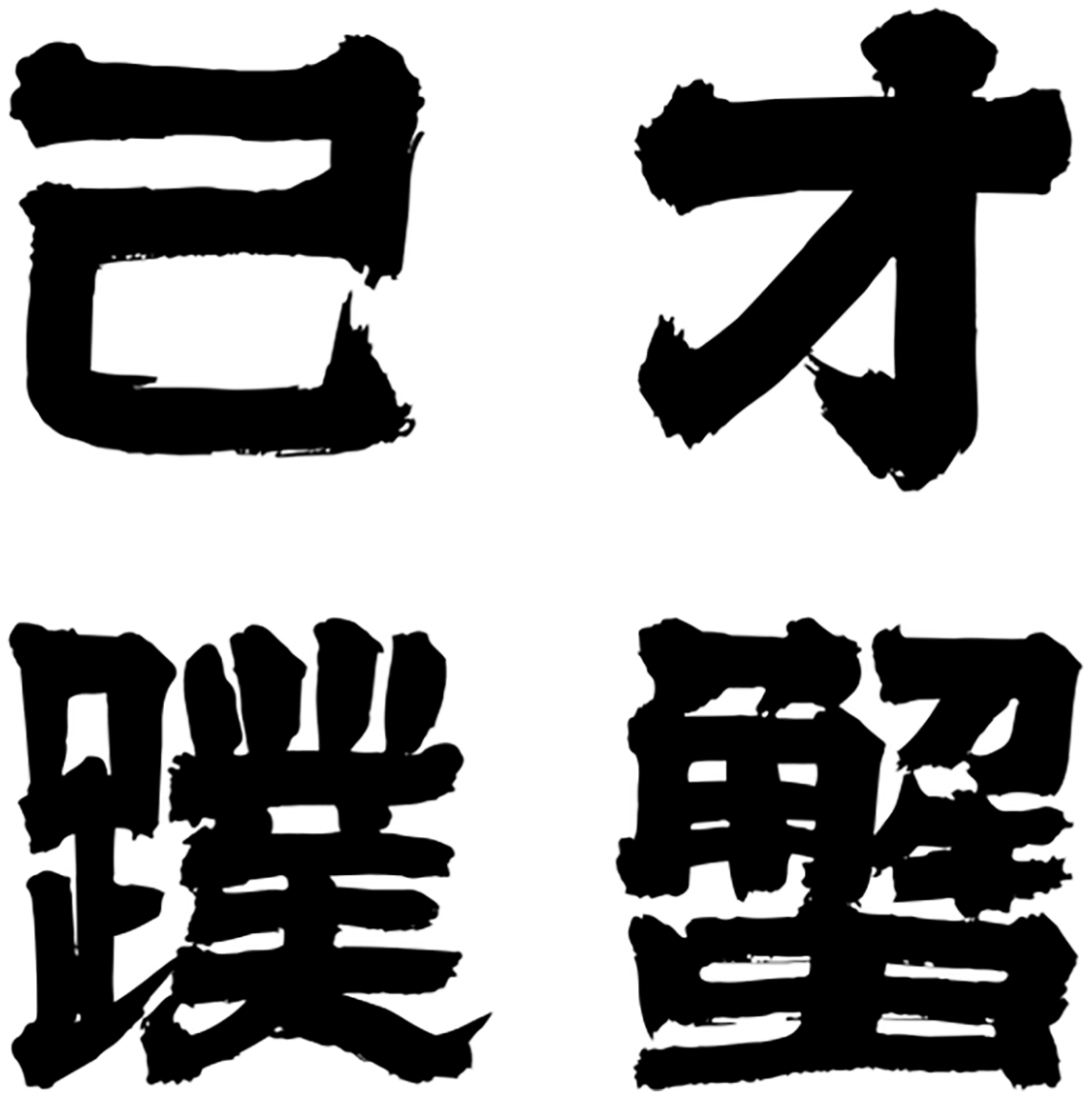
Some examples of selected Chinese characters.

To evaluate the performance of the model proposed in this paper, we created a training and test dataset based on the Founder font library [24] [31], a Chinese font resource containing a wide range of font styles. From this library, we randomly selected 100 font styles and generated 4,000 random Chinese characters for each style, covering nearly all commonly used fonts. We used 80% of these fonts for training and the remaining 20% for testing. Each font character has a resolution of 96×96. We uniformly randomly sampled 4,000 characters from the list of 6,626 Chinese characters provided by [25] as the evaluation set. Some of the characters are shown in Fig 4. The list itself is not sorted by frequency, so random sampling can avoid corpus bias. At the same time, we counted the number of strokes after sampling to confirm that the characters obtained covered the full range from extremely simple structures (≤ 5 strokes) to highly complex structures (≥ 15 strokes), ensuring morphological diversity. In order to input texts with different fonts and Chinese characters, we designed a solution to automatically mark text inputs for specified fonts and Chinese characters. This ensures reproducibility and avoids a lot of time-consuming and laborious manual labeling.

Specifically, we divide the text input into three parts, consisting of three levels: global style, stroke features, and typesetting attributes. The global style is mapped into 10 style labels based on the family name of the font. The stroke features are automatically divided into 10 stroke features using a hard threshold function through statistics of geometric aspects such as stroke width variance and end angle. Finally, 10 typesetting attributes are classified by combining the font measurement and projection histogram. The three-layer label constitutes a fixed structural template sentence, with up to 1,000 random combinations, which meets the requirements of multiple fonts and Chinese characters in the dataset.

In order to quantitatively analyze our model with other models, this paper uses the commonly used evaluation indicators for font transfer: SSIM, RMSE, LPIPS and FID. Structural Similarity Index (SSIM) [19] is usually used to calculate the structural similarity between the generated font and the target font, which is very important for the consistency evaluation of structural character. Root Mean Square Error (RMSE) is commonly used to measure the pixel-level difference between the generated font and the target font, providing a direct quantification of the accuracy between the generated output and the target. Learned Perceptual Image Patch Similarity (LPIPS) [20] is typically used to compute deep features that assess the perceptual difference between the generated font and the target font, aligning more closely with the human visual system. Finally, Fréchet Inception Distance (FID) [21] is commonly used to evaluate the quality and style consistency between the generated font and the target font distribution. In the training phase, $g_{i}^{tar}$ and $g_{i}^{src}$ are input simultaneously and the pixel-perceptual joint loss between the generated result $\hat{g_{j}}$ and the corresponding true value $g_{j}^{tar}$ is calculated; in the inference phase, only $g_{i}^{tar}$ and $g_{i}^{src}$ are needed to synthesize $\hat{g_{j}}$, and $g_{j}^{tar}$ is no longer used; during the evaluation, $\hat{g_{j}}$ is aligned with the retained $g_{j}^{tar}$, and the SSIM, FID and other indicators are counted.

In terms of experimental details, we use the AdamW optimizer to train the model, where the optimizer parameters $\beta_{\text{1}}=0.9$, $\beta_{\text{2}}=0.999$. In the total training rounds, we use a batch size of 16 and a total number of iterations of 300,000. The initial learning rate is set to $1e^{-4}$ and decreases linearly with training to prevent model overfitting. All experiments are completed on a single NVIDIA RTX 3090. Due to the design of deep clipping and time step optimization, the diffusion step size is only 50 steps.

### 4.2 Model performance verification

In terms of comparison with the most advanced models, we selected several advanced methods. First, Stable Diffusion [24] is the most influential text-to-image generation benchmark. It has demonstrated amazing capabilities in decoding text and generating high-resolution images, providing a benchmark reference for the expansion of this task from “text to image” to “text + image to image”. Second, DG-Font (also known as DG-GAN) [23] is a typical font generation method based on generative adversarial networks. Its deformable generative network optimizes the consistency of font shapes, thus providing a benchmark for the generation capability of GAN models. Finally, Diff-Font [13] first introduced the diffusion model into one-shot font generation. It achieved excellent performance through the stroke-level noise prediction strategy, providing a benchmark reference for the input fusion of this task from “image to image” to “text + image to image”. The above three baselines cover the key paradigms from the classic model Diffusion, GAN to the most cutting-edge diffusion model, providing a comprehensive benchmark reference for evaluating the comprehensive performance of our method. Specifically, in RMSE and LPIPS, these two indicators are related to direct pixel calculation and human perception calculation, so the improvement is not obvious compared with other methods. However, in SSIM and FID, two indicators directly related to generation quality and style, our model has obvious advantages. By adding the guidance of pictures and texts to the diffusion process, our model can not only better retain the structural features of the target font, but also make the style of the reference font more consistent with the target font in terms of visual style. Among other methods, whether it is a GAN-based model or a Diffusion-based model, their input is only a single input of text or picture. During the training process, they fail to effectively integrate multiple information to generate fonts. At the same time, the generation method based on GAN lacks stability. Our model guides the diffusion model by fusing text and pictures, so that the model not only accurately focuses on the structure of the target font, but also better matches the target in style and details. Secondly, our model also uses deep pruning and time step optimization improvements, which not only effectively compresses the model’s parameters and reduces the computational complexity, but also allows the model to maintain a high generation quality. In other methods, whether based on GAN or Diffusion models, the number of parameters is very large, especially the Diffusion model as a large model, which is known for its large number of parameters. Due to the large number of parameters, under the same experimental conditions, the training time and inference time are very slow. As shown in Table 1, after our deep pruning and time step optimization, redundant network connection layers are removed, the iteration time step of the model is optimized, and the consumption of computing resources is reduced, making the generation process more efficient, reducing the number of parameters, and reducing the training time and inference time. Finally, the model has improved all indicators, and the number of parameters and training and inference time are reduced.

**Table 1:** Comparison of our model with other state-of-the-art methods.

Model	SSIM↑	RMSE↓	LPIPS↓	FID↓	Parameters↓	Training Time(h)↓	Inference Time (min/100images)↓
Stable Diffusion [24]	0.67	2.71	0.24	12.99	200M	3.2	20
DG-Font [23]	0.69	2.72	0.20	13.68	120M	2.7	23
Diff-Font [13]	0.69	2.71	0.21	13.46	180M	2.5	25
Ours	0.91	2.69	0.07	13.87	100M	1.3	21

**Fig 5:**
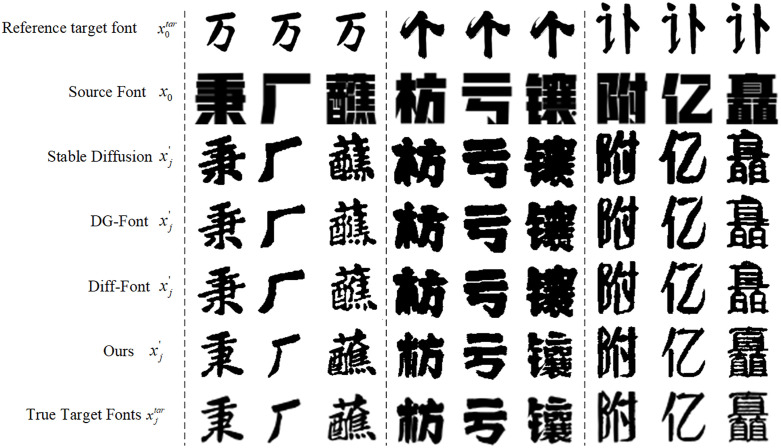
Comparison of the results of our model and other methods. In transferring between different fonts, our model shows significant advantages in various aspects of font transfer.

The different font transfer results generated by our model and the comparison model are shown in Fig 5. Among them, $x_{\text{0}}^{tar}$ corresponds to the style of the target font provided in Fig 1. $x_{\text{0}}$ corresponds to the font skeleton of the target font provided in Fig 1. $x_{j}^{\prime }$ corresponds to the prediction of the diffusion model in Fig 1. $x_{j}^{tar}$ and $x_{j}^{\prime }$ are used to calculate the loss during model training and provide true values during prediction to compare the quality of model prediction results. As shown in the figure, our model shows significant advantages in various aspects of font transfer. In terms of structural integrity, the number of strokes, stroke arrangement, and specific details of complex fonts (such as the third, sixth, and ninth columns) are highly consistent with the target font. In contrast, the fonts generated by Stable Diffusion have missing or sticky strokes, such as in the third and fifth columns. DG-Font has improved in detail expression, but the overall smoothness leads to a lack of realism, such as in the sixth column. Diff-Font is more stable than Stable Diffusion for complex fonts, but details are still missing, such as the eighth column in the second column.

In terms of style consistency, there is a high degree of style consistency between our font and the target font. On the second, fourth, and eighth columns, our results are almost exactly the same in terms of stroke thickness. The results of Stable Diffusion are rough and have a single style. The strokes of DG-Font are inconsistent in thickness and do not look realistic enough. Diff-Font mimics the font style to a certain extent, but it is slightly lacking in the transition of complex strokes, such as the third, sixth, and ninth columns.

In terms of topological structure and clarity, our fonts are clearer and more delicate. For example, the left-right structure of the fourth and sixth columns and the top-bottom structure of the ninth column are restored very accurately, and the restoration resolution is also higher. However, DG-Font and Diff-Font have slight deformation in complex structures and the spacing between strokes is not completely restored. Stable Diffusion performs poorly in topology and clarity.

**Fig 6:**
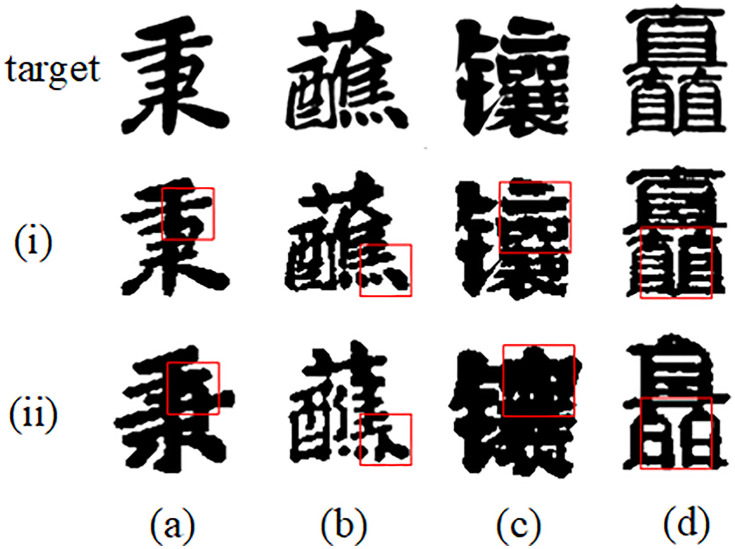
Differences between extreme values of different indicators.

In order to show the difference between extreme values of different indicators, we compare the images that are more different from the target image with the images we generated, as shown in Table 1 and Fig 6. In Fig 6, we use image (i) (better) and image (ii) (worse) as paired examples. In Fig 6(a), the SSIM of Figure (i) is 0.91, while that of Figure (ii) is only 0.71, indicating that the latter has obvious stroke adhesion and breakage, and the structural consistency is significantly reduced; in Fig 6(b), the RMSE of the two images are 3.26 and 3.33 respectively. The overall brightness difference is very small, but the local lack of ink is diluted by the average pixel, resulting in only a slight increase in the value; the LPIPS given in Fig 6(c) surges from 0.11 to 0.32, revealing that a large area of white space in Figure (ii) is filled with ink blocks, texture and contrast are lost, and the distance from the true value in the perceptual feature space is significantly widened; finally, in Fig 6(d), the FID of the single approximate calculation increases from 32.2 to 32.6, indicating that both images are far away from the true distribution, and further distortion will only bring limited increments, but FID needs to be calculated on the entire test set, and the single image is only for reference. The red framed parts are the obvious differences between the two images. In summary, SSIM can best reveal skeleton defects, RMSE mainly reflects global brightness shift, LPIPS is extremely sensitive to texture and stroke differences, and FID is more suitable for evaluating the distribution consistency of the entire set of samples. In general, the improved model has better smoothness in detail accuracy, style consistency and generation stability, overcoming the limitations of the previous font transfer model and showing better performance.

### 4.3 Ablation experiments

In this section, we will conduct several ablation experiments to demonstrate the effectiveness of our proposed model. For text encoding, we use BERT as a comparison, and for image encoding, we use ResNet for comparison. The comparison results of their combined models are shown in Table 2. BERT is a powerful text encoder, which is often used to understand text in complex contexts. However, in the font transfer task, its help for font generation is relatively limited, and it cannot capture the relationship between text prompts and image generation well. The text encoding part of CLIP is specially designed for multimodal tasks. It can well understand the potential relationship between text and image, so its encoding can provide better help for font generation. In the image encoder, ResNet is a classic convolutional neural network. It adds residual connections to solve the gradient vanishing problem in deep networks, but it mainly captures local features and lacks attention to global information. ViT can globally model the information in the image through the self-attention mechanism, not only capturing local information, but also capturing global dependencies, maintaining the consistency of image structure and the richness of details. After various combinations of them, the combination of CLIP, which can provide text and image understanding, and ViT, which captures local and global information, as the guiding module has the best performance.

**Table 2:** Performance comparison of different text and image encoders.

Bootstrapping Model	Text Encoder	Image encoder	SSIM	RMSE	LPIPS	FID
CLIP+ViT	CLIP	ViT	0.91	2.68	0.07	13.87
BERT+ViT	BERT	ViT	0.85	3.58	0.16	15.50
BERT+ResNet	BERT	ResNet	0.82	3.35	0.18	17.33
CLIP+ResNet	CLIP	ResNet	0.80	3.40	0.22	18.79

To verify the effectiveness of the added compression model, we conducted ablation experiments on model compression. From Table 3, we can see that using depth pruning and time step optimization alone can improve the generation performance and generation efficiency compared with the original model, but there are still some shortcomings. Using depth clipping alone mainly reduces the depth of the diffusion model, reducing storage and computational costs. However, due to the reduction in depth, some features are lost in the diffusion model and the generated quality is reduced. Although using time step optimization alone can effectively reduce the number of sampling times, the original model itself has not been changed too much and the computational requirements are still very high. When deep layer pruning is combined with time step optimization, the two methods complement each other. Depth pruning optimizes the computational approach while reducing the model diffusion depth. Although there is a small amount of information loss, these losses are almost negligible due to the optimization of the time step. At the same time, these shallower features are more effectively used in the selected time step to give full play to the role of these features. The combination of the two not only significantly reduces the training and inference time of the model, but also avoids the limitations of a single method through various optimizations. Therefore, joint optimization can achieve more efficient and comprehensive improvements while significantly improving model performance.

**Table 3:** The impact of different model compression modules on the model.

Model	Deep Pruning	Timestep Optimization	SSIM	RMSE	LPIPS	FID	Parameters	Training Time(h)	Inference Time (min/100images)
Origin Model	×	×	0.85	3.01	0.16	22.20	140M	3.1	20
Origin Model	×	√	0.87	3.03	0.13	21.50	140M	2.0	27
Origin Model	√	×	0.86	3.02	0.17	15.30	100M	2.3	28
Origin Model	√	√	0.91	2.68	0.07	13.87	100M	1.3	21

We conducted ablation experiments on the selection of the number of depth shear layers in the diffusion model, and the results are shown in Table 4. At the original number of layers, the model performance was weak and the model parameters were large. As the number of layers was continuously sheared, from shearing 1 layer to shearing 3 layers, the performance of the model continued to rise, but after shearing 3 layers, the performance declined, which shows that after shearing too many layers, the diffusion process has an impact on the generation of fonts. Therefore, although shearing 3 layers has the least number of parameters and the shortest training and inference time, it is still not possible to shear 3 layers. In the end, we chose to shear 2 layers.

**Table 4:** The impact of different diffusion model depths on the model.

Pruning Layers	SSIM	RMSE	LPIPS	FID	Parameters	Training Time(h)	Inference Time (min/100images)
Origin U-Net	0.85	3.20	0.15	22.20	150M	2.3	32
Drop 1 Layers	0.88	3.15	0.14	21.50	120M	2.7	28
Drop 2 Layers	0.91	2.68	0.07	13.87	100M	2.3	25
Drop 3 Layers	0.87	3.10	0.13	15.87	80M	1.3	21

On the time step selection of the diffusion model, we performed ablation experiments, and the results are shown in Table 5. At the original time step, the model performance is weaker and both training and inference times are longer. When reducing the original 100 time steps to 50 time steps, the model reaches its best performance, while the training time and inference time also decrease significantly. However, if the time steps are continued to be reduced, the structural consistency and style of the generation will gradually weaken due to insufficient time steps and insufficient generation quality. Finally we choose 50 time steps.

**Table 5:** The impact of different diffusion model time steps on the model.

Timesteps	SSIM	RMSE	LPIPS	FID	Training Time(h)	Inference Time (min/100images)
100	0.85	3.20	0.15	12.20	1.8	28
50	0.91	2.68	0.07	13.87	1.3	21
30	0.88	3.10	0.13	11.92	0.8	13
20	0.86	3.15	0.14	10.17	0.7	12

## 5. Conclusion

Font design needs to maintain beauty and functionality in complex strokes and diverse styles. This requires full use of font transfer technology to fully improve design efficiency. Although the generation method based on large models (such as the Diffusion model) has achieved some results in font transfer tasks, it is very limited in processing complex fonts and computational efficiency. Therefore, a higher quality and more efficient improved Diffusion model is needed. To this end, we propose an improved Diffusion model guided by the fusion of text and images. Specifically, we encode the text input through CLIP text encoding and the image input through ViT image encoding, and input their encoded latent variables into the diffusion process to guide font transfer. At the same time, we introduce deep clipping and time step optimization techniques to reduce the depth of the U-Net in the diffusion model, and remove the time steps that have little impact on the generation in the later time steps, remove redundant calculations, and improve computational efficiency. Experimental results show that our model performs better than existing methods in processing complex font generation, diversified style transfer and stability, showing very high performance and broad application prospects.

## References

[1] Liu P, Guo Y, Wang F, Li G. Chinese named entity recognition: the state of the art. Neurocomputing. 2022;473:37–53. doi: 10.1016/j.neucom.2021.10.101

[2] Wang X, Li C, Sun Z, Hui L. Review of GAN-based research on chinese character font generation. Chin J Electron. 2024;33(3):584–600. doi: 10.23919/cje.2022.00.402

[3] Azadi S, Fisher M, Kim VG, Wang Z, Shechtman E, Darrell T. Multi-content GAN for few-shot font style transfer. CVPR. 2018.

[4] Hassan AU, Memon I, Choi J. Real-time high quality font generation with Conditional Font GAN. Expert Syst Appl. 2023;213:118907. doi: 10.1016/j.eswa.2022.118907

[5] Yang S, Wang Z, Wang Z, Xu N, Liu J, Guo Z. Controllable artistic text style transfer via shape-matching GAN. arXiv (Cornell University). 2019.

[6] Wu L, Chen X, Meng L, Meng X. Multitask adversarial learning for Chinese font style transfer. 2020.

[7] Li W, He Y, Qi Y, Li Z, Tang Y. FET-GAN: font and effect transfer via K-shot adaptive instance normalization. AAAI. 2020;34(02):1717–24. doi: 10.1609/aaai.v34i02.5535

[8] Salimans T, Goodfellow I, Zaremba W, Cheung V, Radford A, Chen X. Improved techniques for training GANs. Adv Neural Inf Process Syst. 2016;29.

[9] Che T, Li Y, Jacob AP, Bengio Y, Li W. Mode regularized generative adversarial networks. In: International Conference on Learning Representations (ICLR); 2017.

[10] Srivastava A, Valkov L, Russell C, Gutmann MU, Sutton C. VEEGAN: reducing mode collapse in GANs using implicit variational learning. Adv Neural Inf Process Syst. 2017;30.

[11] Bie F, Yang Y, Zhou Z, Ghanem A, Zhang M, Yao Z, et al. RenAIssance: a survey into AI text-to-image generation in the era of large model. IEEE Trans Pattern Anal Mach Intell. 2024:1–20. doi: 10.1109/TPAMI.2024.3522305 40030812

[12] Kondo T, Takezaki S, Haraguchi D, Uchida S. Font style interpolation with diffusion models. In: Lecture notes in computer science; 2024. p. 86–103.

[13] He H, Chen X, Wang C, Liu J, Du B, Tao D, et al. Diff-font: diffusion model for robust one-shot font generation. Int J Comput Vis. 2024;132(11):5372–86. doi: 10.1007/s11263-024-02137-0

[14] Thamizharasan V, Liu D, Agarwal S, Fisher M, Gharbi M, Wang O. VecFusion: vector font generation with diffusion. 2022 IEEE/CVF Conference on Computer Vision and Pattern Recognition (CVPR), Vol. 33; 2024. p. 7943–52.

[15] Chen S, Li Z, Liang D. CD-font: one-shot font generation via conditional diffusion model with disentangled guidance. In: Lecture notes in computer science. Springer Nature Singapore; 2024. p. 279–90. doi: 10.1007/978-981-97-5600-1_24

[16] Zhang L, Zhu Y, Benarab AC, Ma Y, Dong Y, Sun J. DP-font: chinese calligraphy font generation using diffusion model and physical information neural network. 2024:7796–804.

[17] Dosovitskiy A, Beyer L, Kolesnikov A, Weissenborn D, Zhai X, Unterthiner T, et al. An image is worth 16x16 words: transformers for image recognition at scale. International Conference on Learning Representations; 2021.

[18] Radford A, Kim JW, Hallacy C, Ramesh A, Goh G, Agarwal S. Learning transferable visual models from natural language supervision. 2021.

[19] Wang Z, Bovik AC, Sheikh HR, Simoncelli EP. Image quality assessment: from error visibility to structural similarity. IEEE Trans Image Process. 2004;13(4):600–12. doi: 10.1109/tip.2003.819861 15376593

[20] Zhang R, Isola P, Efros AA, Shechtman E, Wang O. The unreasonable effectiveness of deep features as a perceptual metric. arXiv:180103924 [cs] [Internet]. 2018. Available from: https://arxiv.org/abs/1801.03924

[21] Heusel M, Ramsauer H, Unterthiner T, Nessler B, Hochreiter S. GANs trained by a two time-scale update rule converge to a local nash equilibrium [Internet]. Available from: https://proceedings.neurips.cc/paper/2017/file/8a1d694707eb0fefe65871369074926d-Paper.pdf

[22] Nichol A, Dhariwal P. Improved denoising diffusion probabilistic models. arXiv (Cornell University). 2021.

[23] Xie Y, Chen X, Sun L, Lu Y. DG-font: deformable generative networks for unsupervised font generation. 2022 IEEE/CVF Conference on Computer Vision and Pattern Recognition (CVPR); 2021.

[24] Rombach R, Blattmann A, Lorenz D, Esser P, Ommer B. High-resolution image synthesis with latent diffusion models. 2022 IEEE/CVF Conference on Computer Vision and Pattern Recognition (CVPR); 2022.

[25] Cheng SI, Chen YJ, Chiu WC, Tseng HY, Lee HY. Adaptively-realistic image generation from stroke and sketch with diffusion model. 2023 IEEE/CVF Winter Conference on Applications of Computer Vision (WACV); 2023.

[26] Zhang L, Rao A, Agrawala M. Adding conditional control to text-to-image diffusion models. 2023.

[27] Zhu Y, Li Z, Wang T, He M, Yao C. Conditional text image generation with diffusion models. arXiv (Cornell University). 2023.

[28] Lu C, Zhou Y, Bao F, Chen J, Li C, Zhu J. DPM-solver: a fast ODE solver for diffusion probabilistic model sampling in around 10 steps [Internet]. arXiv.org; 2022 [cited 2023 Jun 25]. Available from: https://arxiv.org/abs/2206.00927

[29] Jolicoeur-Martineau A, Li K, Piché-Taillefer R, Kachman T, Mitliagkas I. Gotta go fast when generating data with score-based models. arXiv (Cornell University). 2021.

[30] Li Y, Wang H, Jin Q, Ju H, Chemerys P, Fu Y, et al. SnapFusion: text-to-image diffusion model on mobile devices within two seconds. arXiv (Cornell University). 2023.

[31] Founder Type. FindFont [Internet]. Beijing: Founder Type; [cited 2025 Mar 17]. Available from: https://www.foundertype.com/index.php/FindFont/index

